# Child Life in past Times

**Published:** 1955-11

**Authors:** S. W. Hinds

**Affiliations:** Lecturer in Child Health and Social Medicine, University of Bristol


### CHILD LIFE IN PAST TIMES

BY

S. W. HINDS, M.D., M.R.C.P.

*Lecturer in Child Health and Social Medicine, University of Bristol*

Though ageless aeons have subtly and unobtrusively drawn an impenetrable veil over every detail of family life of prehistoric man, abundant information exists to suggest that the weak and defenceless members of the civilizations of early historic times were often exploited and suffered much at the hands of their fellows. The inexorable struggle to live and the necessity of preserving a healthy verile stock probably dictated their attitude to their weaker brethren. Many peoples including the Spartans, Celts and Romans, for instance, required all newly born infants to submit to searching scrutiny to exclude evidence of poor development and physical defect. Some even imposed rigorous tests of fitness, as for example the survival of total immersion in cold water shortly after birth. Infants who failed these and other tests automatically forfeited the right to live, and were either killed outright or abandoned to die of exposure and starvation.

A fearful belief in theocratic influence and power did nothing to ease the burdens of infancy, and countless babies and young children were in addition mutilated, starved, abandoned or done to death in desperate efforts to conciliate wrathful gods, these rituals taking a higher toll of girls than boys.

Standards of child care in these early communities showed wide variation. The matriarchal civilizations of Babylon, Egypt, India and China treated their children well, but later, a change to patriarchal supremacy saw a wide and rapid spread of infanticide with universal child slavery in India, whilst the child in China was compelled to become absolutely subservient to the adult.

In Rome and Greece children suffered from tyrannical rêgimes until the advent of Christian teaching exulted parenthood, and the Romans then emerged the first people to introduce special legislation for the communal care of unwanted and deserted children. Elsewhere the Hebrews under strict moral and religious codes consistently lavished care on their young, and the Talmud describes their ancient protective ceremony of "salting" which invariably preceded the swaddling of their newborn.

### CHILDREN IN BRITAIN IN EARLY TIMES

The evidence suggests that the ancient Britons, like so many early peoples, placed little value on life and even less on child life, frequently sacrificing their young to appease their gods. Excavations at Woodhenge have, for example, brought to light the remains of a ritual or dedicatory ceremony, in the shape of an infant's skeleton with the skull neatly cleft in two. As witness of such practices Julius Caesar wrote

"The men have power of life and death over their wives and children."

The early phases of the Roman occupation brought little change. Young Britons were shipped in numbers as slaves to Mediterranean countries, the practice persisting until Constantine proclaimed Christianity the official creed of the empire, and outlawed infanticide, child cruelty and slavery. Safe from adult exploitation the young natives enjoyed the benefits of Roman culture and, with many parents adopting the toga, children were admitted to the schools established by the invaders.

Improvements were, however, but short-lived, for with the forced withdrawal of the Roman garrisons the islanders found themselves defenceless against the furious and relentless onslaughts of hordes of heathen Angles and Saxons. The Anglo-Saxon conquest expunged every vestige of Christian thought except where the mountainous and inhospitable terrain of the North and west kept the invaders out, and once more barbaric practices with all their former ferociousness struck terror into the hearts of children, the aged and the infirm.

As the sixth century drew to a close a tiny flame of hope was kindled by the evangelical teachings of St. Columba (565) in the north, and St. Augustine (597) in the south. In due time the flame cast its light over the islanders and invaders alike, uniting in the fold of Christ the haughty and the humble, the conquerors and the conquered, and within the next hundred years, Christian kings were drawing up laws to safeguard child life.

> "Let VI shillings be paid for the fostering of a foundling for the first year; XII shillings the second; XXX shillings the third; afterwards according to its appearance." (Ine of Wessex, A.D. 688–725.)
>
> "If anyone commit his infant to another's keeping, and he die during such keeping, let him who feeds him prove himself innocent of treachery, if anyone accuse him aught." (Alfred, A.D. 871–901.)

In the wake of conversion, monastic houses sprang up, offering on an ever-increasing scale, succour and shelter to all unfortunates including orphaned and abandoned children, while many accepted responsibility for all infants orphaned within their precincts. Beda, writing in the eighth century recorded

> "There was in the same monastry a boy not above three years old called Esica; who by reason of his infant age was bred up among the Virgins dedicated to God, and there to pursue his Studies."

A few establishments were opened solely to shelter children, as was St. Leonard's in York founded by Stephen in 1135, where a woman was specially appointed to look after a number of orphaned boys.

### THE MIDDLE AGES

By the Middle Ages it had become the custom of the monasteries to provide food and shelter for everyone in need. Among many seeking help were numerous children, infirm and aged persons, and large numbers of healthy men "sturdy beggars" who, as the monks gratuitously disbursed alms to all who asked without distinction, saw in the monasteries an easy and effortless means of life.

The closure and spoliation of these houses between 1527 and 1534 threw all these people into the streets without hope of help from any quarter. Forming into bands, they roamed the countryside, preying on the inhabitants of small towns, villages and lonely farmhouses, wreaking havoc and spreading terror wherever they went.

> "Hark, hark, the dogs do bark,
> The beggars are come to town,
> Some in rags, some in jags and some in velvet gown."

Vagrancy became so pressing a problem that methods to control it became urgent. At first a number of "hospitals" were opened for the crowds of wandering homeless children, among them Christ's Hospital in Newgate Market, London, and similar institutions in Bristol in 1586 and Totnes in 1589. These measures, however, scarcely affected the crisis and in 1601 the Poor Law Act was passed expressly to rid the community of vagrancy. Relief was provided from taxes levied in the parishes, and workhouses were built to accommodate all those requiring "indoor" as distinct from "outdoor" relief.

Though a large proportion of those needing "indoor" relief were infants and very young children, no facilities for their care were provided in the workhouses. They were therefore passed quite indiscriminately to any woman willing to foster them for a small fee, a system which not only attracted women of the baser sort, many of them frank criminals, but initiated a spate of infant suffering and cruelty probably never exceeded in history.

As every infant was an added burden on the parish, and workhouse overseers, mindful of the hatred with which the Poor Law taxes were received by the people, resorted to inhuman practices to keep numbers down. Hunting down unmarried mothers and extorting sums of money from putative fathers for the care of "the cow and the calf", they hustled the girls across the boundary into an adjoining parish, even late on in labour, and to ensure that few infants survived many were handed to women who had acquired the reputation of "excellent killing nurses".

The few children who survived returned to the workhouses in early youth often to be apprenticed to established criminals or notorious rogues, who used their apprentices, as did many who professed to be chimney sweeps, to help them in their nefarious activities. Throughout the country thousands of other parish boys and girls were shipped in droves to the mines and later the mills and the factories.

This was an age when prosperity and plenty flourished along with starvation, cruelty and abject squalor. Coarseness and brutality predominated, life was cheap, and society allowed many to sink to levels of human degradation rarely experienced by mankind. In the homes of the poor, tumbledown, draughty shacks, or dark, dank, vermin-infested cellars, in the workhouses bare and comfortless, and in the narrow, twisting, sewage-strewn streets groping aimlessly from the towns to blend unnoticed with deeply rutted muddy country lanes linking the villages, crimes of violence against defenceless infants and children, their very defencelessness being the cause of their downfall, were committed both by day and by night.

"The court at Hecks Hall lately committed Ann Martin, alias Chapney, to Newgate where she is to be imprisoned for two years pursuant of her sentence; she is accused of putting out the eyes of children with whom she went abegging about the country. She has been several times whipped at the carts' tail."

"Elizabeth Banks at the Old Bailey for stripping and robbing in Marylebone Fields an infant aged four."

Children were hanged for petty offences, sometimes for stealing a crust or a few pence to purchase food, while the homeless and destitute were taken into custody, and deported, a procedure deemed to be slightly less drastic than capital punishment.

"That such children who shall be apprehended and are afflicted with any disease and have no persons to take care of them, be cured of such disease, and then transported."

Childbirth, a most hazardous event, was believed to give the mother in normal labour an equal chance of survival if she did not perish from child-bed fever. The advice and assistance of swineherds and shepherds was sought in complicated labours and Germany actually forbade herdsmen attending mothers in 1580. Three centuries later, Clark still described midwives in 1815 as "women usually of the most ignorant types" and later Dickens immortalized them in the character of Mrs. Gamp. The newborn were commonly given gin, opiates and empirical concoctions, such as Godfrey's cordial or Dalby's carminative to still their crying, relieve "the wind" and assist "teething", while parents abandoned large numbers of infants, and thousands more were overlaid, a procedure which came to be recognized as a respectable method of ridding oneself of an unwanted child. How many infants and children perished will never be known, but in 1662 John Graunt (1620–74) a London haberdasher interested in the events going on around him, attempted to estimate the loss of child life by examining the London Bills of Mortality. Publishing his observations in book form he observed:

"In twenty years there dying of all diseases and casualties 229,250; that 71,124 died of the Thrush, Convulsions, Rickets, Teeth and worms and as abortives, chrysoms, and livegrown overlaid—that is to say about one third of the whole died of those diseases, which we guess did all light upon children under 4 or 5 years old."

Almost a century later, Michael Underwood concluded no improvements had occurred. Of all christened children, between 1730 and 1750—for the only records of births were the baptismal entries in church registers, and most unwanted infants were never baptised—75 per cent. died before their fifth birthday. Few appear to have regarded the enormous infant death-rate as out of the ordinary. For instance, Evelyn wrote in his diary of the loss of his son:

26th March 1664. "It pleased God to take away my sonne Richard, being now a moneth old, yet without any sickness or danger perceivably, being to all appearances a most likely child; we suspected much the nurse had overlayne him; to our extreme sorrow being now again reduced to one; but God's will be done."

29th March 1664. "After evening prayers was my child buried neere the rest of his Brothers—my very deare children."

And Gibbon, in his memoirs, epitomized the small chance a child had of surviving in his observation

"The death of a new born child before that of its parents may seem an unnatural but it is a strictly probable event."

### THE EIGHTEENTH CENTURY

Space will not allow more than a passing reference to the guild apprenticeship system, the employment of children in the mines, the mills and the factories, the climbing boys, early paediatricians, and the many people who by their efforts, often sacrifices, individually and collectively over the centuries helped the cause of children forward in many different ways.

Two men, however, deserve special mention for their courage, perseverance in the face of great odds, and the manner in which they influenced their fellow citizens at a time when a child's life was not considered worthy of a thought.

*Thomas Coram and the London Foundling Hospital* (1668–1751).

Probably the earliest foundling hospital was that established in Milan in A.D. 787 by Datheus, archbishop of that city, others following in Venice in 1380 and in Florence in 1421. Some two centuries later Vincent de Paul organized a movement in France to prohibit the wilful mutilation of infants for begging purposes, and opened a number of homes to shelter destitute children.

In Britain after the dissolution of the monasteries and the failure of the special "hospitals" to visibly reduce the numbers of indigent children on the streets, the Poor Law system, which created issues greater than those it was designed to alleviate, assumed responsibility for every unwanted infant and child.

In 1719, in the autumn of his days, Thomas Coram, a Dorset man who had spent all his life closely associated with the sea, retired to live quietly in Rotherhythe, a small hamlet on the lower Thames. In the evenings he delighted in strolling along the river banks to the outskirts of London, when he noticed enormous numbers of infants, some already dead, others moribund, exposed and abandoned in the gutters and on dung heaps. Much affected by these sights he resolved to open a home for starvelings. He spent the next seventeen years trying to raise funds for his project, and eventually obtaining the signatures of twenty-two "ladies" in 1738, the London Foundling Hospital opened its doors on 25th March 1741 to "nineteen females and eleven males, being all it could contain". Initially only infants under two months old were accepted, who after baptism were sent to nurses in the country, returning to London at the age of four for lessons in reading, writing and the scriptures. Later they were apprenticed with honest and reliable guildsmen. Applications for admission rose steeply, more than one hundred and sixteen infants being offered in a single day in 1753. Three years later with the hospital in acute financial difficulties, a successful appeal was made to Parliament for support. It was then decided to accept all unwanted children. The response was immediately overwhelming; mothers clamoured and fought on the doorstep to have their children admitted, and the workhouses transferred all their young inmates. Other infants were sent by road from all parts of the country, some travelling more than three hundred miles. Many perished *en route*, while countless others never left their home towns alive, the carriers murdering them once the fare had been paid. In all, fifteen thousand infants were accepted in four years, of whom ten thousand died. Shocked by the results, Parliament withdrew its support and the hospital reframed its eligibility regulations.

These events so excited public opinion that many eminent personalities of the day became interested in the Hospital. Handel raised money by giving performances of

*Messiah*, on one occasion selling tickets to the value of seven hundred guineas, while Reynolds, Benjamin West and Wilson arranged public exhibitions of their works. Churchmen, including Bishop Maddox, preached most eloquently on child life, while Hogarth influenced public opinion with his brilliant satyrical pictures of aspects of life.

### *Jonas Hanway* (1712–86) *and the Parish Child*

At the time when Coram's hospital was struggling to take every infant it could manage, and the public was aware to some extent of the vile and inhuman ways in which illegitimate and pauper children were handled by their parents and society, few raised their voice in dissent, until Jonas Hanway published his findings in 1757 on the perilous plight of the parish child.

"Of one important parish which in fourteen years did not preserve a single child."

"Out of fifty-three being the whole number received in five years, not one was kept alive."

"Parish of St. Clement Dane—twenty three children sent to Mary Pool, ten died within the year"—and of St. Luke, Middlesex and St. James Westminster "the discharged and dead balanced the born and received."

Estimating the death-rates among workhouse children to be in the vicinity of eighty-ninety per cent. rising to ninety-nine per cent. in infants under a year old, he was instrumental in obtaining the passage of the Act of 1767, quickly to be dubbed "The Act for keeping children alive."

A growing revulsion against the hideous acts of hate and cruelty levelled against defenceless, indigent childhood was soon discernable abroad, and by the close of the century many parishes had already redrawn their fostering regulations. For instance, the parish of St. James Westminster was by 1797 placing their foundlings with selected and well-supervised women living on Wimbledon Common, at a fee of 3*s.* 0*d.* a week. Among other innovations they introduced a bonus of a guinea payable if a child lived a year and a further five shillings should it recover from measles.

### THE MATERNITY AND CHILD WELFARE MOVEMENT

By the middle of the nineteenth century the public was stirring to the special needs of mothers and children. Lying-In hospitals and hospitals for children were opened, while the universities created chairs in midwifery and diseases of women. At the same time men and women, both medical and lay, had begun to consider the impact of the environment and the community on family and child life, and though ideas crystallized in two or three different ways it was not until many years later that it was realized the several independent movements were each striving for the same objects. With the realization, the Maternity and Child Welfare Movement came into being.

At the suggestion of William Farr (1807–83) the London Obstetrical Society investigated the infant deaths for the years 1867–69 and shewed that though seventy-five per cent. of the labours had been conducted by midwives, not one of them had been "instructed", i.e. had been trained. This finding was followed in 1870 by the demand that

"No person should be allowed to perform the duties of midwife or accoucheur unless he or she shall have received proper instruction."

In spite of the unassailability of this demand, nevertheless women had to wait until 1902 before the Midwives Act, after many vicissitudes, eventually reached the Statute Book, and even then a clause allowed untrained midwives to continue practice until 1910.

In that year 1870, the Infant Protection Society was formed following a growing dissatisfaction of the standards of care of most foster-children. Helped by the trial and execution of Margaret Waters, a notorious "baby farmer", the Society was successful in obtaining the passage of the Child Life Protection Act of 1872.

About this time too, with the infant mortality rate showing no evidence of falling though the birth-rate was declining, for instance, it moved from 35·5 per cent. between 1871–75 to 29·3 per cent. between 1896–1900, interest in childhood began to grow.

These developments initiated further critical inquiry into the probable causes of the annual epidemics of gastroenteritis which were taking such a heavy toll of infant life. The findings all pointed to the home and indicated poor levels of home hygiene, ignorance, and low standards of maternal care, results which at once led to the formation of voluntary organizations to carry the newer views on infectious disease, personal and home hygeine, home management and mothercraft to the homes of the people.

Among the organizations was one, the Ladies Sanitary Reform Society formed in Manchester in 1862, which was destined to play an outstandingly important role in the next fifty years. By 1890 Manchester Corporation fully recognized the invaluable work of these women volunteers drawn from all walks of life and invited them to co-operate with the corporation under the aegis of the Medical Officer of Health, an invitation they readily accepted, to establish the first Municipal Welfare Service in Britain.

By the early years of the present century Manchester had sixteen visitors on their staff, some voluntary and others whole-time salaried municipal workers who, between them, were carrying out some 1,000 to 1,500 home visits a year. So successful did this system prove that by 1905 over fifty other local authorities had inaugurated similar services, calling their visitors by many different titles, amongst them " sanitary inspectors " and " health visitors".

It was not now long before all those interested in mothers and children recognized that they were basically striving independently for the same ends, and the realization matured with the passage of the Maternal and Child Welfare Act, of 1918. Further consolidation followed with the publication of the Health Visitors' Training Regulations by the Board of Education.

The problem of a high infant mortality rate with a falling birth-rate was also one that faced France in the second half of the last century. Tackling it from another angle, Pierre Budin (1846–1907) in 1882 established at the Charitê Hospital, Paris, a "Consultation de Nourissons" where advice on infant feeding was offered only to mothers delivered in that hospital, but two years later in 1894, Dr. Leon Dufour opened a "Goutte de lait" at Fecamp, with the object of stimulating a general interest in infant feeding and especially to encourage breast feeding and disseminate knowledge on hygienic feeding at home.

The scheme became popular overnight and rapidly spread through the country even to beyond its boundaries, and five years later in 1899 a Milk Depot was opened in St. Helens, Lancs. It provided safe and suitable artificial feeds for infants of all ages and took every opportunity to encourage natural feeding. By 1901, Ashton under Lyme, Liverpool and Dunkenfield had opened similar depots, and by 1905 many others were offering advice and guidance on mother and baby craft.

That year the first international conference on infant welfare was held in Paris, attracting delegates from twenty-two countries. No official British representative was present, but the few who attended privately, convinced at the value of the meeting were, on their return to Britain, instrumental in having the first international gathering on infant mortality meet in London in 1906 under royal patronage.

In 1914 the Local Government Board proposed making grants available to advance maternal and child welfare work, in a scheme by which both local authorities and voluntary bodies could qualify for assistance, and at the same time the decision was taken to include the pre-school child. Other proposals, including several Acts of Parliament, notably the Notification of Births (Extension) Act of 1915, helped the movement gain ground over the war years 1914–18, and the number of clinics rose from 650 in 1915 to 1,278 by 1918.

From these several sources and through innumerable vicissitudes, the Maternity and Child Welfare Service has grown and matured, incorporating new ideas as they have arisen, its sole object being to improve the health and happiness of mothers and children and the conditions in the homes and families to which they belong. In Britain today this comprehensive service is accepted by the public without question as the natural heritage of all infants and young children, though few realize how massive a gap has been bridged in less than a century. The very helplessness of childhood which caused its undoing and let it fall victim to the basest and vilest sides of man's nature, is today the keystone bonding together the many services, voluntary and statutory, which have evolved that the child may enjoy the right to live, grow and develop in a happier, healthier and better enlightened environment.

